# Postmortem diagnosis of pulmonary tumor thrombotic microangiopathy with rapid exacerbation in a patient with gastric cancer: a case report

**DOI:** 10.1186/s12245-021-00377-2

**Published:** 2021-09-15

**Authors:** Ryo Kamidani, Keisuke Kumada, Hideshi Okada, Genki Yoshimura, Tomohiro Kanayama, Hiroyuki Tomita, Tomotaka Miura, Hideaki Oiwa, Yosuke Mizuno, Yuichiro Kitagawa, Ryu Yasuda, Tetsuya Fukuta, Takahito Miyake, Tomoaki Doi, Takahiro Yoshida, Shozo Yoshida, Akira Hara, Shinji Ogura

**Affiliations:** 1 Advanced Critical Care Center, Gifu University Hospital, 1-1 Yanagido, Gifu, 501-1194 Japan; 2 Department of Tumor Pathology, Gifu University Graduate School of Medicine, Gifu, Japan; 3 Abuse Prevention Center, Gifu University Graduate School of Medicine, Gifu, Japan

**Keywords:** Pulmonary tumor thrombotic microangiopathy, Pulmonary hypertension, Tumor microembolism, Oncologic emergency

## Abstract

**Background:**

Pulmonary tumor thrombotic microangiopathy (PTTM) is a condition that involves the development of pulmonary hypertension due to the presence of microscopic tumor emboli of the peripheral pulmonary arteries. Here, we report a case of rapidly exacerbating PTTM associated with gastric cancer that was identified postmortem through pathological autopsy.

**Case presentation:**

A 52-year-old Asian woman who experienced anterior chest pain while coughing visited the orthopedic department of the Gifu University Hospital. She was diagnosed as having multiple osteolytic bone metastases throughout her body and was subsequently scheduled to undergo combined positron emission tomography and computed tomography (CT) to search for a primary lesion. However, 4 days after her visit to the orthopedic department, she was unable to stand up and thus visited the emergency department. At the time of admission, physical examination results revealed that she had a percutaneous oxygen saturation level of 90% (on room air) and cyanosis and that she was in a state of hemodynamic shock. Laboratory test results revealed elevated levels of fibrin degradation products and D-dimer in her blood. Chest CT results were normal. She was admitted to the hospital’s general ward for follow-up but soon entered a gradually worsening state of shock and respiratory failure. Electrocardiography revealed findings associated with right heart strain; however, contrast-enhanced CT did not reveal the presence of pulmonary embolism. She was admitted to the intensive care unit and was treated for pulmonary hypertension; however, 45 h after her arrival at the hospital, she died of respiratory failure. A pathological autopsy revealed the presence of gastric cancer, tumor microemboli, and fibrous intimal thickening of the peripheral arteries of both lungs; thus, a diagnosis of PTTM was made.

**Conclusions:**

In patients with carcinoma of unknown primary site and pulmonary hypertension with pulmonary embolism ruled out by CT, emergency physicians and intensivists must consider the possibility of PTTM, which represents an oncologic emergency, and initiate chemotherapy administration as soon as possible.

## Background

Pulmonary tumor thrombotic microangiopathy (PTTM) is a condition associated with pulmonary hypertension due to tumor microemboli in the peripheral pulmonary arteries, leading to rapidly progressive pulmonary hypertension and respiratory failure [1]. Diagnosing PTTM early is difficult because the imaging results of patients with PTTM do not typically reveal lung field abnormalities or the presence of major thrombi or emboli in pulmonary arteries [2]. Death often occurs shortly after the onset of symptoms in patients with PTTM. Here, we report a case wherein a patient’s pathological autopsy revealed the presence of PTTM associated with gastric cancer.

## Case presentation

**Fig. 1:**
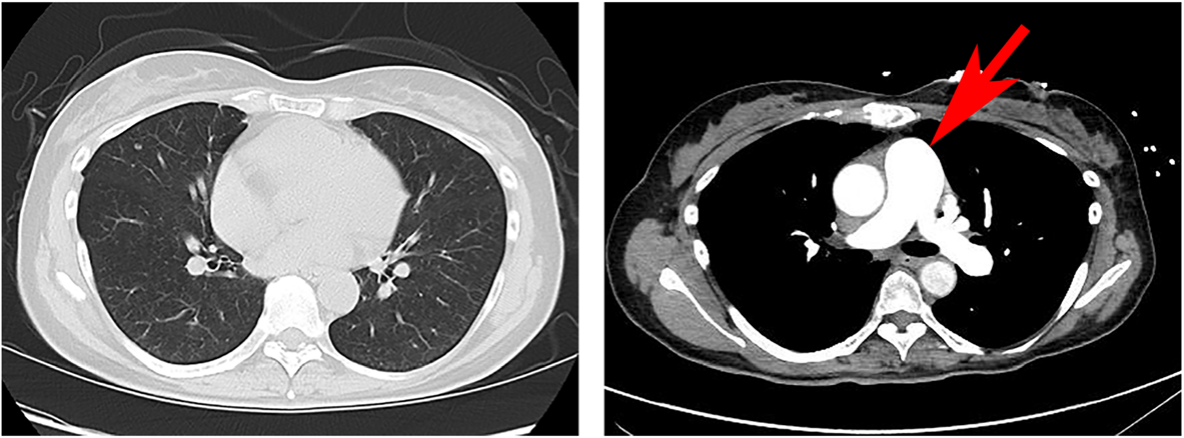
Computed tomographic (CT) images obtained after the patient’s arrival and admission to the hospital. Left panel: thoracic CT image obtained after the patient’s arrival to the hospital shows no abnormal lesion. Right panel: thoracic contrast-enhanced CT obtained after the patient’s admission to the hospital shows no massive pulmonary thromboembolism or tumor emboli. The red arrow indicates aorta enlargement

A 52-year-old woman presented to her family doctor with a 20-day history of anterior chest pain while coughing. A full-body computed tomography (CT) scan revealed abnormal shadowing in her sternum. After 16 days from her visit to the family doctor, she visited the orthopedic department of the Gifu University Hospital and was diagnosed as having osteolytic bone metastasis and partial sclerosis in the sternum, 11th thoracic vertebra, right 4th lumbar vertebra, right femoral head, and left iliac bone. She was scheduled to undergo combined positron emission tomography and CT imaging at a later date. However, 4 days after her visit to the orthopedic department, she was unable to stand up. When she visited the emergency department of our hospital, a physical examination revealed that she had a respiratory rate of 15 breaths/min, blood pressure of 88/43 mmHg, body temperature of 36.3 °C, and heart rate of 116 beats/min. Furthermore, on room air, she had cyanosis and a percutaneous oxygen saturation level of 90%. She was also found to be in a state of hemodynamic shock without the use of catecholamine agonists. Her heart sounds were regular, and no heart murmurs were detected. In addition, her breath sounds were normal, and no rales were noted. An arterial blood gas test was performed (Table 1). Laboratory test results revealed elevated levels of hepatobiliary enzymes, fibrin degradation products (37.7 μg/mL; normal level: ≤ 4.0 μg/mL), and D-dimer (4.6 μg/mL; normal level: <1.0 μg/mL) (Table 1). Electrocardiography revealed only sinus tachycardia. The results of chest radiography and plain chest CT were normal (Fig. 1).

**Table 1:** Laboratory findings at the time of admission

**<CBC>**	
WBC	8350 /uL
RBC	5.36 × 10^6^ /uL
Hemoglobin	14.4 dL
Hematocrit	53.4 %
Platelet	20.0 × 10^4^ uL
**<Coagulation status>**	
APTT	24.8 sec
PT-INR	0.99
Fibrinogen	252 mg/dL
D-dimer	7.1 ug/mL
FDP	37.7 ug/mL
AT III	107 %
**<Venous blood gas>**	
F_I_O_2_	0.21
pH	7.405
PaCO_2_	27.6 mmHg
PaO_2_	70.3 mmHg
HCO_3_^-^	16.9 mmol/L
Base excess	−5.9
Lactate	51 mg/dL
**<Biochemistry>**	
Total protein	7.3 g/dL
Albumin	4.3 g/dL
Creatinine kinase	81 g/dL
Aspartate transaminase	35 IU/L
Alanine transaminase	25 IU/L
Lactate dehydrogenase	316 IU/L
Alkaline phosphatase	869 IU/L
Cholinesterase	390 IU/L
γ-Glutamyl transpeptidase	26 IU/L
Creatinine	0.89 mg/dL
Blood urea nitrogen	12 mg/dL
Total bilirubin	0.7 mg/dL
Sodium	137 mEq/L
Potassium	4.3 mEq/L
Chlorine	99 mEq/L
C-reactive protein	0.55 mg/dL
Blood glucose	223 mg/dL
Hemoglobin A1c	5.8 %

**Fig. 2:**
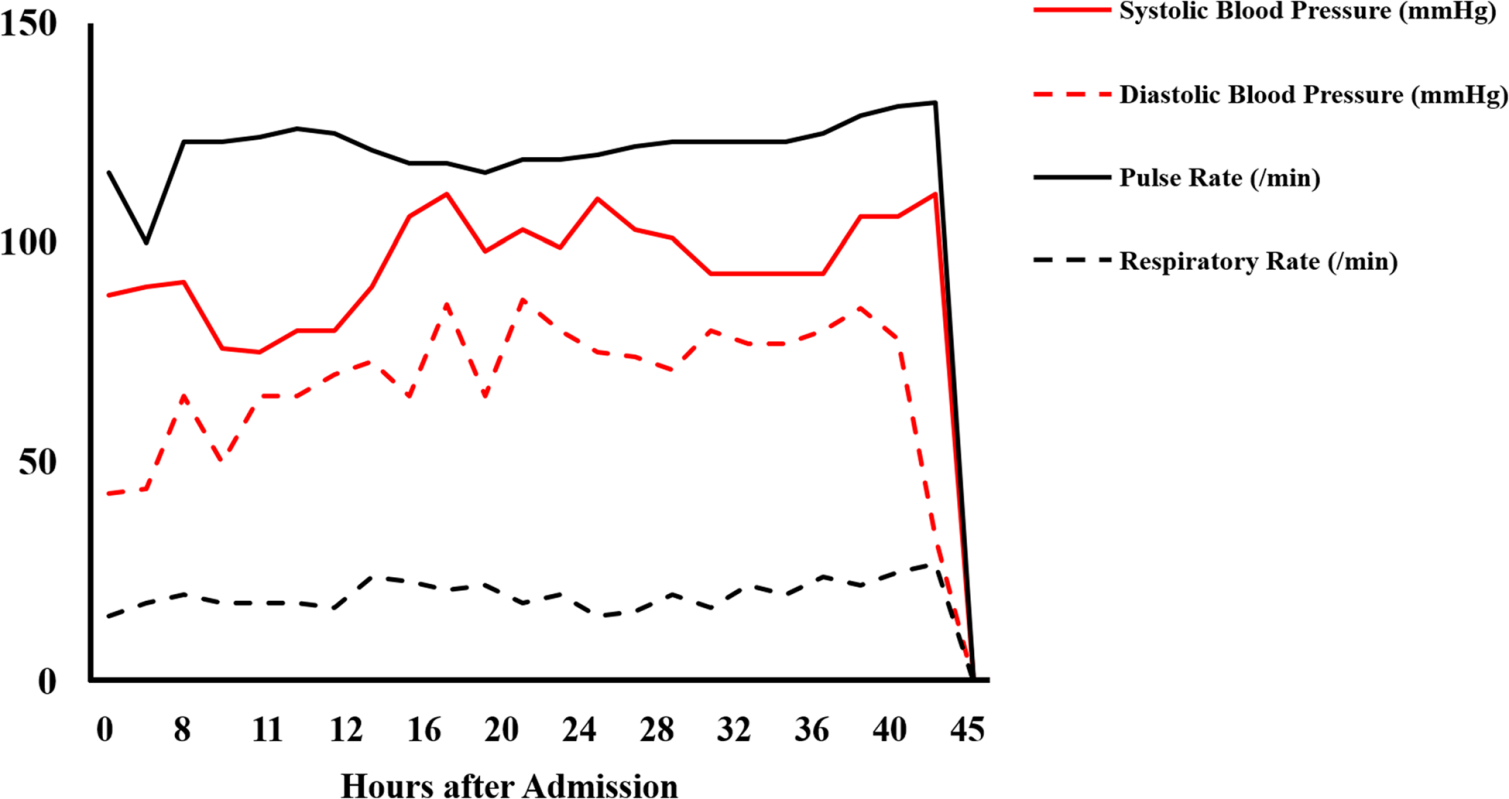
Clinical course of the patient. Changes in systolic blood pressure (mmHg), diastolic blood pressure (mmHg), pulse rate (/min), and respiratory rate (/min) after admission are shown

**Fig. 3:**
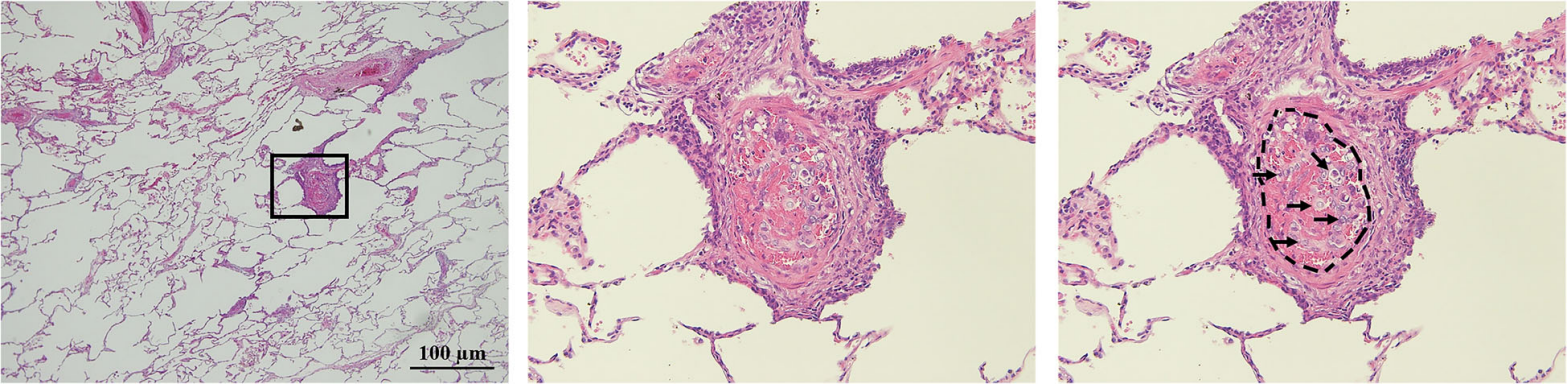
Images obtained during the pathological autopsy performed in the reported case. Left panel: low-magnification image of the right upper lobe. Middle panel: enlarged image of the black square in the left panel showing the presence of fibrous intimal thickening in the peripheral pulmonary arteries. Right panel: guided image of the middle panel. Black arrows indicate cancer cells, and the black dotted line indicates vascular intima

She was then admitted to the general ward for examination and follow-up. Soon after her admission, she went into a state of shock (systolic blood pressure of 70 mmHg) and developed respiratory failure; thus, we initiated oxygen administration. Two-dimensional transthoracic echocardiography revealed findings associated with right heart strain; however, contrast-enhanced CT did not reveal the presence of any obvious thrombus in the pulmonary arteries. Although she was receiving intensive care and was started on treatment for pulmonary hypertension, she died of respiratory failure 45 h after arrival to our hospital (Fig. 2). A pathological autopsy revealed that the patient had gastric cancer with lymph node metastasis around the aorta, stomach, and pancreas and bone metastases in the lumbar vertebrae. The autopsy revealed that the patient had no major tumor emboli; however, the presence of tumor microemboli and fibrous intimal thickening in the peripheral pulmonary arteries were detected (Fig. 3). In addition, intestinal ischemia and the presence of microbleeds in the liver and cerebellum were noted. Ultimately, through postmortem histopathological diagnosis, the patient was determined to have PTTM.

## Discussion

This case report describes a patient with carcinoma of unknown primary site who died because of rapidly progressive dyspnea and hypoxemia and was ultimately diagnosed with PTTM after her death. Therefore, emergency physicians should consider the possibility of PTTM, which represents an oncologic emergency, in patients who present with carcinoma of unknown primary site and pulmonary hypertension and for whom pulmonary embolism has been ruled out.

PTTM was first described in 1990 by Von Herbay et al. [1] as a life-threatening disease associated with severe respiratory failure with rapidly progressive pulmonary hypertension. Unlike pulmonary tumor embolism, PTTM is characterized by fibrous intimal thickening of the peripheral pulmonary arteries, particularly the small arteries. Clinically, PTTM is difficult to differentiate from pulmonary embolism. PTTM is often identified through pathological autopsies and is most commonly associated with gastric carcinoma [2]. Other types of primary cancer that have been reported to be complicated by PTTM include breast cancer, tongue cancer, hepatocellular carcinoma, colorectal cancer, and prostate cancer [3]. Godbole et al. [3] analyzed 160 unique cases and reported the following prevalence rates for predominant PTTM symptoms: hypoxemia, 95%; dyspnea, 94%; abdominal pain, 86%; cough, 85%; and general pain, 73%. In most reported cases of PTTM, elevated D-dimer levels are noted, which makes it even more difficult to distinguish PTTM from pulmonary embolism [4]. Moreover, radiological (chest CT) findings for cases of PTTM are nonspecific and include the presence of centrilobular nodules, ground-glass opacities, linear branching opacities, and interlobular septal thickening [5–8].

Upon admission to the hospital, the patient had a mildly elevated D-dimer blood level. However, chest CT did not reveal any significant findings, and we could not make a definitive diagnosis before the patient’s death. Through the pathological autopsy performed in this case over a wide area of both lungs, we observed the presence of arterial occlusions due to microthrombi and tumor emboli that were present in pulmonary arterioles, accompanied by congestion and hemorrhage. Fibrous intimal thickening of the pulmonary arteries was also noticeable, and this finding is typically associated with PTTM rather than pulmonary embolism.

Although the pathogenesis of PTTM remains unclear, it is considered that an activation of the coagulation system and the release of inflammatory mediators lead to the formation of microthrombi and fibrous intimal thickening of small arteries, which in turn results in the progression of pulmonary hypertension. Furthermore, the congregation of macrophages around the blood vessels and cell-to-cell signaling via osteopontin and CD44 is speculated to contribute significantly to the pathogenesis of PTTM [2]. As mentioned previously, PTTM progresses rapidly. The average duration from onset to hospital admission for PTTM cases is approximately 1 month. In fatal cases, the median survival time is only 5 days [9]. Therefore, PTTM is often diagnosed after the patient’s death, and there are only a few reported cases of PTTM that were diagnosed and treated while the patients were still alive. Pulmonary microvascular cytologic evaluation of samples drawn through a wedged pulmonary artery catheter is the most reasonable diagnostic method when the patient is still alive. The sensitivity and specificity of this technique range from 80–88% and 82–94%, respectively [10, 11].

A unique case of PTTM involved a patient who survived for 7 months after receiving imatinib, in addition to chemotherapy for signet-ring cell carcinoma [12]. Imatinib is a platelet-derived growth factor receptor-tyrosine kinase inhibitor that has the potential to cause reverse remodeling due to its proliferation-inhibitory, apoptosis-inducing, and vasoconstrictive effects. Several other cases involving the use of imatinib for the treatment of PTTM-associated pulmonary hypertension have been reported, suggesting that imatinib is effective for the treatment of not only the primary tumor, but also pulmonary hypertension [13–15]. According to the comprehensive clinical classification system for pulmonary hypertension of the European Society of Cardiology and the European Respiratory Society, pulmonary arterial hypertension is in group 1 [16]. Upfront combination therapy, including treatment with diuretics, prostacyclin analogs, endothelin receptor antagonists, and phosphodiesterase type 5 inhibitors, is recommended for patients with class IV pulmonary hypertension (according to the World Health Organization’s functional classification system for pulmonary hypertension), which is often observed in intensive care units (this therapy should be considered for patients with class IIa disease and may be considered for patients with class IIb disease). However, pulmonary hypertension related to tumor embolism is included in group 5 of the comprehensive clinical classification system. To the best of our knowledge, no randomized controlled trials evaluating the efficacy of drugs for the treatment of pulmonary hypertension associated with tumor embolism have been performed thus far [16]. Other drugs such as corticosteroids and anticoagulants are easier to introduce and have been used in many cases; however, clear effects have not been observed [11, 17, 18].

## Conclusion

As there is no standard diagnostic approach for PTTM, emergency physicians and intensivists should consider PTTM, in addition to pulmonary embolism, if there is a sudden worsening of the respiratory status of a patient who has carcinoma of unknown primary site and pulmonary hypertension. If the patient indeed has PTTM, there is an oncologic emergency to address. If a patient is suspected of having PTTM, it is necessary to initiate the administration of chemotherapy as soon as possible according to the etiology of PTTM. In addition, it is important to continue to search for the primary cancer site in the patient.

## Data Availability

The datasets used and/or analyzed during the current study are available from the corresponding author on reasonable request.

## Abbreviations

PTTM: Pulmonary tumor thrombotic microangiopathy
CT: Computed tomography

## References

[1] von Herbay A, Illes A, Waldherr R, Otto HF (1990). Pulmonary tumor thrombotic microangiopathy with pulmonary hypertension. Cancer.

[2] Price LC, Wells AU, Wort SJ (2016). Pulmonary tumour thrombotic microangiopathy. Curr Opin Pulm Med.

[3] Godbole RH, Saggar R, Kamangar N (2019). Pulmonary tumor thrombotic microangiopathy: a systematic review. Pulm Circ.

[4] Zhang M, Zhang Y, Pang W, Zhai Z, Wang C (2019). Circulating biomarkers in chronic thromboembolic pulmonary hypertension. Pulm Circ.

[5] Tateishi A, Nakashima K, Hoshi K, Oyama Y, Ebisudani T, Misawa M, Aoshima M (2019). Pulmonary tumor thrombotic microangiopathy mimicking inhalation lung injury. Intern Med.

[6] Franquet T, Giménez A, Prats R, Rodríguez-Arias JM, Rodríguez C (2002). Thrombotic microangiopathy of pulmonary tumors: a vascular cause of tree-in-bud pattern on CT. AJR Am J Roentgenol.

[7] Johkoh T, Ikezoe J, Tomiyama N, Nagareda T, Kohno N, Takeuchi N, Yamagami H, Kido S, Takashima S, Arisawa J (1992). CT findings in lymphangitic carcinomatosis of the lung: correlation with histologic findings and pulmonary function tests. AJR Am J Roentgenol.

[8] Kunimatsu A, Kunimatsu N, Yasaka K, Akai H, Kamiya K, Watadani T, Mori H, Abe O (2019). Machine learning-based texture analysis of contrast-enhanced MR imaging to differentiate between glioblastoma and primary central nervous system lymphoma. Magn Reson Med Sci.

[9] Fujishiro T, Shuto K, Shiratori T, Kono T, Akutsu Y, Uesato M, Hoshino I, Murakami K, Imanishi S, Tochigi T, Yonemori Y, Matsubara H (2013). A case report of pulmonary tumor thrombotic microangiopathy (PTTM) caused by esophageal squamous cell carcinoma. Esophagus.

[10] Keenan NG, Nicholson AG, Oldershaw PJ (2008). Fatal acute pulmonary hypertension caused by pulmonary tumour thrombotic microangiopathy. Int J Cardiol.

[11] Miyano S, Izumi S, Takeda Y, Tokuhara M, Mochizuki M, Matsubara O, Kuwata H, Kobayashi N, Kudo K (2007). Pulmonary tumor thrombotic microangiopathy. J Clin Oncol.

[12] Kubota K, Shinozaki T, Imai Y, Kario K. Imatinib dramatically alleviates pulmonary tumour thrombotic microangiopathy induced by gastric cancer. BMJ Case Rep. 2017. 10.1136/bcr-2017-221032. 10.1136/bcr-2017-221032 PMC5589033 28882938

[13] Fukada I, Araki K, Minatsuki S, Fujino T, Hatano M, Numakura S, Abe H, Ushiku T, Iwase T, Ito Y (2015). Imatinib alleviated pulmonary hypertension caused by pulmonary tumor thrombotic microangiopathy in a patient with metastatic breast cancer. Clin Breast Cancer.

[14] Higo K, Kubota K, Takeda A, Higashi M, Ohishi M (2014). Successful antemortem diagnosis and treatment of pulmonary tumor thrombotic microangiopathy. Intern Med.

[15] Minatsuki S, Miura I, Yao A, Abe H, Muraoka H, Tanaka M, Imamura T, Inaba T, Maki H, Hatano M, Kinugawa K, Yao T, Fukayama M, Nagai R, Komuro I (2015). Platelet-derived growth factor receptor-tyrosine kinase inhibitor, imatinib, is effective for treating pulmonary hypertension induced by pulmonary tumor thrombotic microangiopathy. Int Heart J.

[16] Galiè N, Humbert M, Vachiery JL, Gibbs S, Lang I, Torbicki A, Simonneau G, Peacock A, Vonk Noordegraaf A, Beghetti M, Ghofrani A, Gomez Sanchez MA, Hansmann G, Klepetko W, Lancellotti P, Matucci M, McDonagh T, Pierard LA, Trindade PT, Zompatori M, Hoeper M, ESC Scientific Document Group (2016). 2015 ESC/ERS guidelines for the diagnosis and treatment of pulmonary hypertension: the Joint Task Force for the Diagnosis and Treatment of Pulmonary Hypertension of the European Society of Cardiology (ESC) and the European Respiratory Society (ERS): endorsed by: Association for European Paediatric and Congenital Cardiology (AEPC), International Society for Heart and Lung Transplantation (ISHLT). Eur Heart J.

[17] Kayatani H, Matsuo K, Ueda Y, Matsushita M, Fujiwara K, Yonei T, Yamadori I, Shigematsu H, Andou A, Sato T (2012). Pulmonary tumor thrombotic microangiopathy diagnosed antemortem and treated with combination chemotherapy. Intern Med.

[18] Miyazaki S, Ikeda T, Ito G, Inoue M, Nara K, Nishinaga Y, Hasegawa Y (2017). Pulmonary tumor thrombotic microangiopathy successfully treated with corticosteroids: a case report. J Med Case Rep.

